# IMPACT OF EARLY-LIFE EXPERIENCES AND CAREGIVING ON COGNITIVE HEALTH

**DOI:** 10.1093/geroni/igae098.0785

**Published:** 2024-12-31

**Authors:** Karen Graham

**Affiliations:** Chair

## Abstract

This symposium comprises four studies investigating the impact of adverse childhood experiences (ACEs) and caregiving on cognitive health. In the first study, researchers investigate the association between ACEs and cognition in older African Americans, exploring the moderating role of active coping. While ACEs were not directly associated with global cognitive functioning, a negative relationship was found between active coping and cognition. This underscores the need for further research to understand the complex interplay between ACEs, coping mechanisms, and cognitive health. In the second study, researchers explore the association between ACEs and dementia, using data from the Health and Retirement Study. They found that individuals reporting ACEs have significantly higher odds of dementia, especially among men. This underscores the importance of trauma-informed interventions to reduce dementia onset. The third study focuses on data from the National Longitudinal Study of Youth. It reveals significant associations between childhood parental love and affection, dysfunctional parental relationships, and self-rated memory in older adults. Mediation analysis emphasizes the role of psychological resources in shaping subjective cognition. The fourth study examines the long-term effects of ACEs on mental health among Black dementia family caregivers. Results highlight the detrimental impact of childhood trauma on depression, depressive symptoms, and suicidal thinking. These findings emphasize the need for targeted interventions to mitigate the mental health consequences of ACEs in caregiving contexts. Together, these studies provide valuable insights into the multifaceted relationships between early-life experiences, caregiving, and cognitive health, highlighting the importance of targeted interventions to support individuals across the lifespan.

